# Disposal of expired medicines in Jordan: Practices of community pharmacists

**DOI:** 10.1371/journal.pone.0348951

**Published:** 2026-05-15

**Authors:** Rania Hamed, Deemah Hasan H. Hasan, Alaa Hammad, Ameerah Hasan Ibrahim

**Affiliations:** Department of Pharmacy, Faculty of Pharmacy, Al-Zaytoonah University of Jordan, Amman, Jordan; University of Petra (UOP), JORDAN

## Abstract

The handling of expired medicines in community pharmacies in Jordan is a critical issue that warrants greater attention. It impacts the environment, public health, and society. However, no studies have investigated the practices of community pharmacists in Jordan regarding the disposal of expired medicines. Therefore, this study aimed to evaluate the current practices of community pharmacists in Amman, Jordan, regarding the disposal of expired medicines and to assess the measures currently implemented to reduce the number of disposed medicines. A cross-sectional study was conducted among community pharmacists in Amman, Jordan, over three months, from November 2023 to January 2024. A 10-minute questionnaire was distributed either in a paper-based format or via an online platform. Data were analyzed using IBM SPSS version 26. The Chi-square test was used to assess the association between participants’ demographics and their responses to the questions. Of the 353 community pharmacists surveyed, 70.2% (n = 248) were under 40 years old, and 63.5% (n = 224) were female. The majority of the participating community pharmacists reported disposing of solids (n = 227, 64.5%), liquids (n = 206, 58.7%), semisolids (n = 213, 60.7%), and controlled drugs classified as Class B (n = 266, 76.2%) and Class C (n = 253, 72.5%) by returning them to pharmaceutical distributors. However, 75.4% (n = 267) reported a lack of knowledge about the disposal methods used by distributors for expired medicines. Most community pharmacists (n = 191, 57.5%) reported that supplements, including vitamins, minerals, and probiotics, were the most frequently expired products. To reduce expired medicines, community pharmacists most frequently relied on medical representatives to collect near-expiry items (n = 136, 38.5%) or limited stock (n = 125, 35.4%). Most community pharmacists (n = 248, 70.3%) reported awareness of the harmful effects of improper disposal of expired medicines on the environment. The study also found that 195 out of 353 community pharmacists (55.1%) had not received any course on the disposal methods of expired medicines during their pharmacy education. The present study highlighted the critical need for community pharmacists to be knowledgeable about proper disposal methods of expired medicines and to raise awareness of the establishment of specialized centers for their disposal.

## Introduction

Medication waste refers to “any pharmaceutical product that is unused, partially used, or not fully consumed at any stage of the pharmaceutical supply and use chain,” including expired medicines and unused prescriptions [1]. Medication waste is one of the leading causes of pollution worldwide [2]. Previous study reported that improper disposal of expired medicines from pharmacies, households, or patients can release chemicals into the environment via water or soil [3], leading to contamination and antibiotic resistance [4]. Additionally, improperly discarded medication may become accessible to vulnerable populations such as children and individuals at risk of medication misuse or abuse [5,6].

According to the World Health Organization’s (WHO) guidelines, there are seven proper methods for disposing of expired medicines [7]. These methods include returning expired medicines to the manufacturer and landfilling expired medicines [8], immobilizing expired medicines (encapsulation and inertization) [7], flushing expired medicines into sewers [7], burning expired medicines in open containers [9], incineration [10], and chemical decomposition of expired medicines [11] (S1 Table). Whereas, improper disposal methods of expired medicines include throwing them in the garbage or flushing them down toilets or sinks, particularly for dosage forms other than liquids [3].

As the need for pharmaceutical care increases, community pharmacists are increasingly regarded as integral healthcare providers [12]. For instance, community pharmacists play a vital role in the pharmaceutical supply chain and are on the front lines for managing expired medicines [13,14]. Worldwide, community pharmacists often lack knowledge of the proper disposal methods of expired medicines [15]. For instance, a study conducted in Kuwait showed that community pharmacists’ disposal practices were suboptimal, with only 23 of 144 pharmacists (~16%) following the Ministry of Health guidelines [16]. Similarly, in Trinidad and Tobago, a study found that although a large percentage of community pharmacists (80.8%) disposed of expired medicines by returning them to pharmaceutical distributors, a considerable proportion (32.3%) still discarded them in the household trash [14].

The widespread availability of pharmaceutical products can pose environmental risks if community pharmacists do not dispose of them properly at their pharmacies [17]. A previous cross-sectional study in Jordan found that many Jordanians dispose of unused or expired medicines in harmful ways, such as throwing them in trash cans without considering the potential consequences [18]. Therefore, the proper disposal of expired medicines by community pharmacists at their respective pharmacies is a critical issue to address to protect human health and the environment [4]. To date, no studies have investigated the practices of community pharmacists in Jordan regarding the disposal of expired medicines.

Therefore, this study aimed to evaluate the current practices of community pharmacists in Amman, Jordan, regarding the disposal of expired medications and to assess the measures currently implemented to reduce the number of expired medicines. Such an evaluation is essential for developing strategies to reduce the generation of expired medicines in the future and for raising awareness among community pharmacists about the importance of proper disposal practices for both health and environmental protection.

## Methods

### Study design and sampling technique

An observational cross-sectional study was conducted between November 2023 and January 2024 to evaluate the practices of community pharmacists in Amman, Jordan, regarding the disposal of expired medications and actions taken to reduce medication waste. A snowball sampling technique was used to recruit community pharmacists across Amman [19]. A snowball sampling technique was employed to recruit community pharmacists practicing in Amman. This approach was selected due to the lack of an accessible, comprehensive sampling frame of practicing pharmacists and the logistical constraints associated with reaching pharmacists across different geographic regions within the capital. Initial participants “seeds” were identified through professional networks and direct visits to community pharmacies. These pharmacists were then asked to share the survey with eligible colleagues working in other community pharmacies. Recruitment continued iteratively until the target sample size was achieved.

According to the Jordan Pharmacists Association (JPA), there are 1,771 pharmacies in the capital, Amman [19]. The large number of community pharmacies in Amman contributes to the prevalence of expired medicines, making the handling of expired medicines a vital area of research. Additionally, Jordan has an estimated 3,600 community pharmacists practicing in Amman. The minimum recommended sample size for this study was determined using the Raosoft sample size calculator [20]. The minimum sample size was calculated to be 348, with a 95% confidence level and a 5% margin of error.

**Fig 1:**
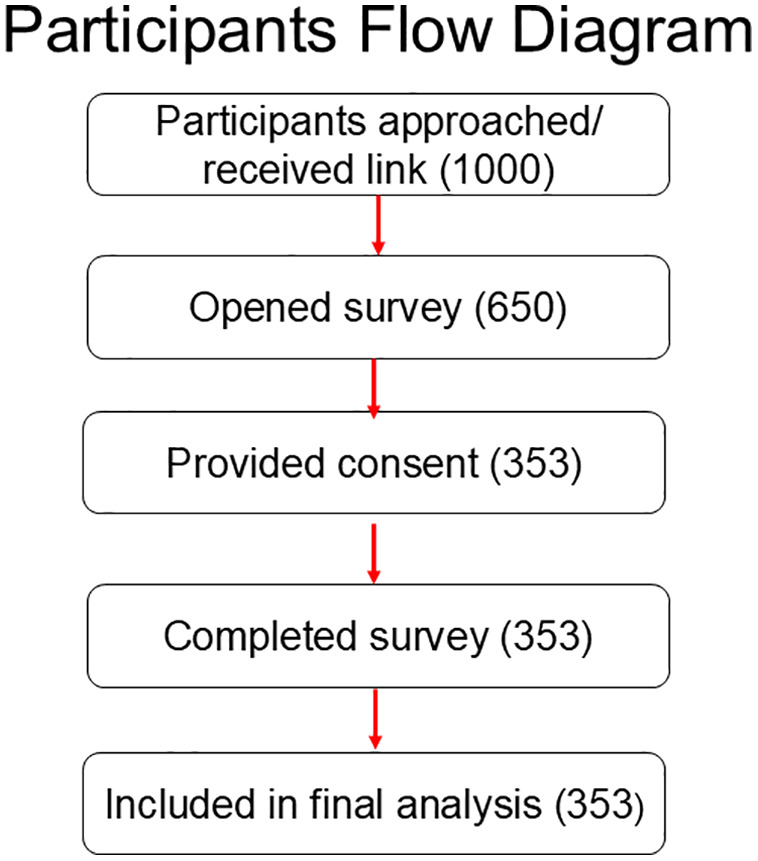
A flow diagram of the study participants who received links.

The questionnaire was distributed to multiple groups of community pharmacists via social media platforms. A flowchart for the study participants who received links is shown in Fig 1.

### Inclusion and exclusion criteria

The study population consisted of practicing pharmacists of all academic degrees, including bachelor’s (BSc), doctor of pharmacy (PharmD), and higher degrees such as master’s and doctor of philosophy (PhD), who had worked in community pharmacies in Amman, had provided informed consent, and agreed to participate. Informed consent was obtained electronically before participants could access the questionnaire. On the first page, participants were provided with information about the study and asked whether they agreed to participate; only those who selected ‘I agree to participate’ could proceed to the survey. Pharmacy assistants or trainees were excluded from this study because they are unlicensed pharmacists.

### Data distribution

Community pharmacists’ knowledge, attitudes, and practices regarding the disposal of expired medicines in Amman. A 10-minute questionnaire was used to collect the data. The questionnaire was adapted from previous studies conducted in other countries, including New Zealand [21], the United Arab Emirates (UAE) [17], and Kuwait [16]. The complete questionnaire was piloted with 20 community pharmacists across various regions of Amman to identify any potential confusion or unclear questions. They were asked for their comments and feedback on the questionnaire, and their responses were used to refine its content and estimate the time required to complete it. During the pilot phase, the participating community pharmacists did not find any questions unclear. The questions were then translated from English to Arabic using the forward-backward-forward technique, as Arabic is the official language of Jordan.

The questionnaire (S2 Table) was structured into four sections and developed in accordance with the Knowledge, Attitudes, and Practices (KAP) framework. The first section collected demographic characteristics of community pharmacists, including age, gender, years of professional practice, pharmacy location, and educational level. It also comprised the knowledge domain, which assessed pharmacists’ awareness of proper disposal methods of expired medicines, potential environmental risks associated with improper disposal, and the availability of specialized disposal systems. The second section focused on the types and forms of expired medicines encountered in community pharmacies, and the disposal methods used. Participants were asked to specify whether expired medicines were in solid, liquid, or semisolid dosage forms, and whether they included controlled drugs classified as Class B (e.g., amphetamines and barbiturates) or Class C (e.g., benzodiazepines and piperazines). The third section explored pharmacists’ attitudes and perceptions regarding the future management of expired medicines in community pharmacies in Amman. It included questions addressing the existence of a dedicated disposal center in Jordan, the perceived need for establishing such a center, and attitudes towards potential funding sources for its establishment and operation (e.g., government, pharmaceutical companies, or community pharmacies). The fourth section assessed current practices implemented by community pharmacists to manage and minimize the accumulation of expired medicines in community pharmacies, including disposal approaches and strategies to reduce the generation of expired medicines.

The questionnaire was mandatory, requiring participants to respond to all items before submission. Data were collected using both paper-based and online questionnaires. Paper questionnaires were distributed during direct visits to community pharmacies in different districts of Amman and were completed on-site. Online questionnaires were disseminated through professional networks and pharmacist communication channels. Participants were encouraged to share the survey link with colleagues, consistent with the snowball sampling strategy. No financial or material incentives were provided for participation. Moreover, there was no missing data in the questionnaires analyzed in the final analysis. This is because the questionnaire was designed to be obligatory, requiring participants to answer all questions before submitting it.

Additionally, the knowledge and attitude items were primarily structured as dichotomous (two-choice, yes/no) questions, without predefined correct or incorrect answers, in line with the descriptive nature of the KAP approach as recommended by WHO guidelines. Therefore, a composite scoring system could not be applied to these domains. In contrast, participants’ overall practice scores were calculated by summing responses to questions 9–13 (range: 0–5), and subsequently categorized into “good” practice (scores ≥3) and “poor” practice (scores <3); this cut-off (≥70% of the maximum score) was predefined to reflect an adequate level of practice. Moreover, Cronbach’s alpha was calculated for these questions to assess the internal consistency of the practice scores [22].

### Ethical issues

The study protocol received approval from the Institutional Review Board (IRB) Committee of the Faculty of Pharmacy at Al-Zaytoonah University of Jordan (IRB Approval number 2/10/2023–2024). Pharmacists provided informed consent by signing the first page of the questionnaire, which describes the purpose of the study. The questionnaire did not include any questions about personal information related to the participating pharmacists or the pharmacies where they work, to ensure their privacy.

### Statistical analysis

All data were exported from Google Forms to Microsoft Excel, coded as needed, and then imported into SPSS version 26 for analysis. Descriptive statistics were performed using IBM SPSS software version 26, along with the Chi-square test, to analyze the data of this study. Cross-tabulation and frequency analysis were conducted on the collected data. Frequency descriptive statistics were utilized for the demographic data. A p-value of ≤ 0.05 was considered significant.

## Results

### Demographic data of the participants’ pharmacists

Table 1 presents the demographics of the participating community pharmacists. Of the 353 questionnaires collected, the demographic data showed that most participating community pharmacists were under 30 years of age (n = 136, 38.4%), followed by those aged 30–39 years (n = 112, 31.6%), indicating that most respondents were younger. Females comprised the majority of participants (n = 224, 63.5%). Additionally, the most significant proportion of community pharmacists had 1–4 years of experience (n = 124, 35.1%). Most participants held a bachelor’s degree in pharmacy (n = 268, 75.9%).

**Table 1:** Demographic data for community pharmacists who participated in the study (n = 353).

	n	%
**Age (years)**		
<30	136	38.4
30-39	112	31.6
40-49	62	17.5
≥50	43	12.2
**Gender**		
Male	129	36.5
Female	224	63.5
**Years of practicing as a pharmacist**		
<1	51	14.4
1-4	124	35.1
5-9	98	27.8
≥10	80	22.7
**Location of pharmacy**		
Center	81	23.1
East	123	35.1
West	146	41.7
**Level of study**		
Bachelor’s in Pharmacy	268	75.9
Bachelor’s in Pharm D	31	8.8
Master’s Degree	47	13.3
Doctor of Philosophy (PhD)	7	2.0

### Disposal methods of expired medicines

#### Disposal methods of expired medicines by community pharmacists.

The proper disposal methods of expired medicines, as outlined by WHO, include returning the expired medicines to the pharmaceutical manufacturer or pharmaceutical distributor, and landfilling them [8], immobilizing the expired medicines by encapsulation and inertization [7], flushing the expired medicines into sewers [7], burning them in open containers [9], incineration [10], or chemically decomposing them [11] (S1 Table). However, the improper disposal methods of expired medicines include throwing them in the household trash or flushing them down toilets or sinks, particularly for dosage forms other than liquids [3]. Table 2 presents the disposal methods of expired medicines used by community pharmacists for expired medicines across various dosage forms in their pharmacies. The majority of participating community pharmacists reported disposing of solids (n = 227, 64.5%), liquids (n = 206, 58.7%), semisolids (n = 213, 60.7%), and controlled drugs classified as Class B (n = 266, 76.2%) and Class C (n = 253, 72.5%) by returning them to pharmaceutical distributors. For solid dosage forms, such as tablets and capsules, the second most common disposal method is discarding them in trash bins (n = 55, 15.6%). In contrast, for liquids (n = 74, 21.1%), semisolids (n = 65, 18.6%), and controlled drugs classified as Class B (n = 58, 16.6%) and Class C (n = 67, 19.2%), the second most prevalent disposal method is flushing them down the toilet.

**Table 2:** Disposal methods used by community pharmacists for expired medicines across various dosage forms in their pharmacies.

	In the rubbish bins		In the sink		In the toilet		Send back to the pharmaceutical distributor		Others	
	n	%	n	%	n	%	n	%	n	%
Solids	55	15.6	5	1.4	48	13.6	227	64.5	17	4.8
Liquids	37	10.5	23	6.6	74	21.1	206	58.7	11	3.1
Semisolids	61	17.5	3	0.9	65	18.6	213	61	7	2.0
Class B	6	1.70	4	1.1	58	16.6	266	76.2	15	4.3
Class C	12	3.40	2	0.6	67	19.2	253	72.5	15	4.3

The “Others” option was included on each question of Table 2 to mitigate forced-choice bias, ensuring that community pharmacists are not required to select a response that doesn’t truly reflect their practice and/or knowledge. One option that community pharmacists might perform under the “Others” is the open burning of expired medicines. Notably, the frequency of “Others” selections was minimal, suggesting that the survey was representative of the participants’ practices and/or knowledge.

A practice score was calculated by summing the adherence across the five categories (solids, liquids, semisolids, Class B, and Class C), reflecting overall compliance with the recommended disposal methods. Higher scores indicate better disposal practices, with a maximum achievable score of 5. The results suggested that while the majority of pharmacists demonstrate good practices, there is still room for improvement, especially in the disposal of liquids and semisolids. A total of 193 participants (54.7%) demonstrated good practice, whereas 160 participants (45.3%) were classified as having poor practice. To ensure the validity and consistency of the practice scores, Cronbach’s Alpha for questions 9–13 was 0.827, exceeding the 0.7 threshold, indicating acceptable internal consistency [23].

#### Handling of expired medicines by distributors.

**Fig 2:**
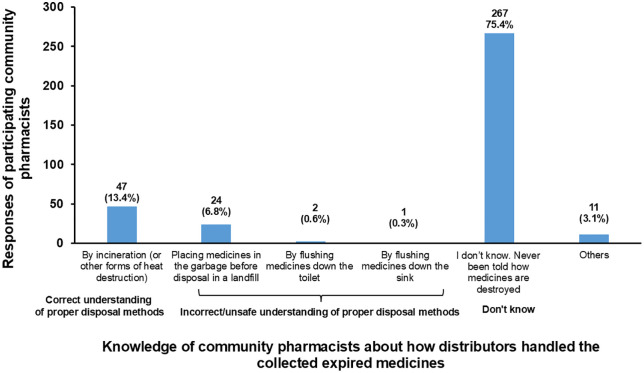
Knowledge of community pharmacists about how distributors handled the collected expired medicines.

Fig 2 presents the knowledge of community pharmacists about how distributors handle expired medicines collected from community pharmacies. Most participating community pharmacists (75.4%) reported being unaware of how distributors ultimately dispose of expired medicines. Among those who indicated specific methods, 13.4% believed that distributors use incineration, which aligns with WHO-recommended disposal methods. However, smaller proportions reported that distributors dispose of expired medicines in garbage before landfill (6.8%) or by flushing them into sinks or toilets (0.9%). It is important to note that flushing and disposal in general waste prior to landfill are considered proper disposal methods according to WHO guidelines. These responses reflect pharmacists’ perceptions rather than verified distributor practices.

### Community pharmacists practice in reducing expired medicines

#### Actions taken by community pharmacists to reduce expired medicines.

**Fig 3:**
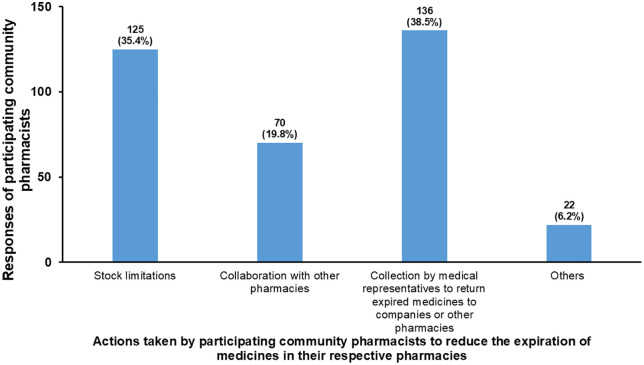
Actions taken by community pharmacists to reduce the generation of expired medicines in their pharmacies.

Fig 3 summarizes the actions taken by participating community pharmacists to reduce medicine expiration in their pharmacies. The most common response from community pharmacists (n = 136, 38.5%) was that medical representatives collect nearly expired medicines from pharmacies, allowing them to be returned to distributors or redistributed to other pharmacies for sale. Another response involved limiting medication stock (n = 125, 35.4%). Collaborating with other pharmacies that are more in need of these nearly expired medicines was the least frequently reported method among participating community pharmacists (n = 70, 19.8%) for reducing medicine expiration in pharmacies. Regarding other actions, some community pharmacists (n = 22, 6.2%) suggested that combining strategies, including stock limitations, collaboration with pharmacies, and the collection of nearly expired medicines by medical representatives, is effective for minimizing medicine expiration. Furthermore, a few community pharmacists (n = 3, 0.8%) proposed that encouraging physicians to prescribe generic names rather than brand names could help reduce medicine expiration.

#### Most commonly expired products in community pharmacies.

**Fig 4:**
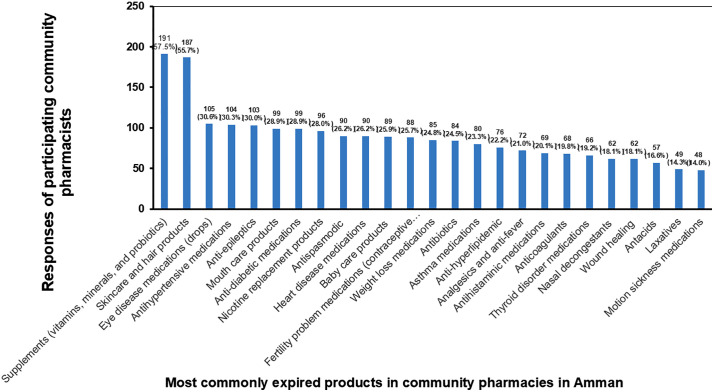
The products most frequently expired in community pharmacies, listed in descending order.

Fig 4 presents the responses of participating community pharmacists regarding the most commonly expired products in community pharmacies in Amman, listed in descending order. Most community pharmacists (n = 191, 57.5%) reported that supplements, including vitamins, minerals, and probiotics, were the most frequently expired products. Additionally, many community pharmacists (n = 187, 55.7%) reported that skincare and hair products ranked second among the most expired items. In contrast, fewer community pharmacists (n = 48, 14.0%) and n = 49, 14.3%) identified motion sickness medications and laxatives, respectively, as the least-expired products.

#### Awareness of community pharmacists on the harmful risks of expired medicines to the environment.

Table 3 presents the community pharmacists’ awareness of the risks associated with the improper disposal of expired medicines. Most of the participating community pharmacists (n = 248, 70.3%) reported awareness of the harmful effects of improper disposal of expired medicines on the environment. The study also found that 195 of 353 community pharmacists (55.1%) had not received any course on the proper disposal methods of expired medicines during their pharmacy education. Furthermore, most participating community pharmacists (n = 290, 82.2%) reported never having attended lectures or workshops organized by health authorities on the proper disposal of expired medicines. Moreover, most community pharmacists (n = 320, 90.7%) expressed the need to participate in future lectures and workshops focused on the proper disposal of expired medicines.

**Table 3:** Community pharmacists’ perceptions of how distributors handle collected expired medicines.

Question asked	n	%
Are you aware of the dangers of improper disposal of expired medicines to the environment or to the health of society?		
Yes	248	70.3
No	105	29.7
As a community pharmacist, have you learned how to dispose of expired medicines in pharmacy schools?		
Yes	158	44.8
No	195	55.2
Have you ever attended any workshops or lectures hosted by health authorities on the proper disposal methods of expired medicines?		
Yes	63	17.8
No	290	82.2
As a community pharmacist, do you think attending workshops or lectures hosted by health authorities is vital to knowing exactly how to dispose of expired medicines?		
Yes	320	90.7
No	33	9.30

### Community pharmacists’ attitudes on the disposal of expired medicines in Jordan

Table 4 presents the community pharmacists’ attitudes regarding the disposal of expired medicines in Jordan. Of the 353 participating community pharmacists, 337 (95.5%) emphasized the urgent need to establish a center dedicated to collecting and properly disposing of expired medicines in Jordan. Additionally, most community pharmacists (n = 248, 74.9%) reported that this specialized center should be funded by health authorities. In contrast, other community pharmacists proposed that pharmaceutical companies (n = 75, 22.7%), patients (n = 6, 1.8%), and community pharmacies (n = 2, 0.6%) should sponsor the center. When asked whether community pharmacies would be willing to pay for the services provided by this specialized center, most community pharmacists (n = 273, 77.3%) answered no.

**Table 4:** Attitudes of community pharmacists toward the disposal of expired medicines in Jordan.

Question asked	n	%
Do you think Jordan needs a specialized center for disposing of expired medicines?		
Yes	337	95.5
No	16	4.50
The funding of this center should be provided by:		
Health authorities	248	74.9
Patients	6	1.80
Pharmaceutical companies	75	22.7
Community pharmacists	2	0.60
Are the community pharmacies willing to pay for this center’s services in disposing of waste medicines?		
Yes	64	19.0
No	273	81.0

#### The association between the demographics of the participating community pharmacists and their practices, knowledge, and attitudes regarding the disposal of expired medicines.

Table 5 presents the association between the age of participating community pharmacists and disposal practices and related knowledge. The results indicated that the age of community pharmacists significantly affected pharmacists’ actions in reducing expired medicines in their community pharmacies (p-value = 0.031). For instance, pharmacists above 30 agreed that limiting medicine stocks could help reduce expired medicines. Whereas pharmacists under 30 believed that reducing expired medicines could be achieved by asking medical representatives to collect near-expiration medicines and return them to their companies or redistribute them to other pharmacies for sale. Regarding disposal methods of expired medicines, the results showed a statistically significant difference (p-value = 0.012) between the two age groups in their preferred methods for disposing of solid, liquid, and semisolid dosage forms.

**Table 5:** Association between age and disposal methods and related knowledge.

		Age				p-value
		<30 (n = 136)		≥30 (n = 216)		
		N	%	N	%	
Routes of disposal of solid medications	In the rubbish bins	13	9.6	42	19.4	0.012
	In the sink	1	0.7	4	1.90	
	In the toilet	18	13.2	30	13.9	
	Send back to the pharmaceutical distributors	101	74.3	126	58.3	
	Others	3	2.2	14	6.50	
Routes of disposal of liquid medications	In the rubbish bins	10	7.4	27	12.6	0.029
	In the sink	6	4.4	17	7.90	
	In the toilet	24	17.6	50	23.3	
	Send back to the pharmaceutical distributors	94	69.1	112	52.1	
	Others	2	1.50	9	4.20	
Routes of disposal of semisolid medications	In the rubbish bins	16	11.8	45	21.1	0.044
	In the sink	1	0.70	2	0.90	
	In the toilet	21	15.4	44	20.7	
	Send back to the pharmaceutical distributors	96	70.6	117	54.9	
	Others	2	1.50	5	2.30	
Actions to reduce the expiration of medications in community pharmacies	Stock limitations	39	28.7	86	39.6	0.031
	Collaboration with other pharmacies to exchange nearly expired medicines	29	21.3	41	18.9	
	Withdrawal by medical representatives to return to companies or redistribute to other pharmacies	63	46.3	73	33.6	
	Others	5	3.70	17	7.80	
Have the pharmacists ever attended workshops or lectures hosted by health authorities on the proper disposal methods of expired medicines?	Yes	14	10.3	49	22.6	0.004
	No	122	89.7	168	77.4	
Knowledge of the presence of a specialized center for handling expired medicines	Yes	12	8.80	37	17.1	0.039
	No	124	91.2	180	82.9	
Attitude on which party should be responsible for the funding of these specialized centers	Health authorities	110	83.3	142	67.6	0.002
	Patients	1	0.80	6	2.90	
	Pharmaceutical companies	19	14.4	61	29.0	
	Community pharmacies	2	1.50	1	0.50	

Additionally, the study revealed a statistically significant association (p-value = 0.004) between the age of participating community pharmacists and their attendance at workshops and lectures on the proper disposal of expired medicines organized by health authorities. The analysis showed that 14 community pharmacists (10.3%) under the age of 30 and 49 community pharmacists (22.6%) above the age of 30 attended workshops or lectures on the proper disposal methods of expired medicines hosted by health organizations. Moreover, when assessing community pharmacists’ awareness of a specialized center for disposing of expired medicines in Jordan, it was evident (p-value = 0.039) that pharmacists under 30 reported that such a center does not exist. Furthermore, the community pharmacists’ attitudes on the funding source for establishing an expired medicines disposal center were significant (p-value = 0.002), with the most frequent responses being the health authorities.

Table 6 presents the association between participating community pharmacists’ gender and their knowledge and attitudes regarding the specialized center in Jordan for the disposal of expired medicines. The study analysis demonstrated a statistically significant association between community pharmacists’ gender and their perception of a specialized center in Jordan for disposing of expired medicines and the need to establish one (p-value = 0.004). Female pharmacists mostly indicated that Jordan lacks such a specialized center and strongly agreed on the need for one.

**Table 6:** Association between the gender and community pharmacists’ knowledge and attitudes regarding a specialized center in Jordan for the disposal of expired medicines.

		Gender				p-value
		Male (n = 129)		Female (n = 224)		
		N	%	n	%	
Knowledge of the presence of a specialized center in Jordan for expired medication disposal	Yes	27	20.9	22	9.80	0.004
	No	102	79.1	202	90.2	
Attitude on the need for a specialized center in Jordan	Yes	118	91.5	219	97.8	0.008
	No	11	8.50	5	2.20	

Table 7 presents the association between community pharmacists’ years of practice and their training and attitudes regarding the proper disposal of expired medicines. The study analysis revealed that community pharmacists’ years of practice significantly affected their responses. For instance, community pharmacists with less than 4 years of practice (n = 175, 49.7%) had not attended workshops or lectures on the proper disposal of expired medicines (p-value = 0.012). Additionally, community pharmacists with less than 4 years of practice (n = 139, 80.8%) reported that a specialized center for the disposal of expired medicines should be funded by health authorities (p-value = 0.009).

**Table 7:** Association between years of practice and community pharmacists’ training and attitudes regarding the disposal of expired medicines.

		Years of practice				
		<4 years		≥4 years		p-value
		n	%	n	%	
Have the pharmacists ever attended workshops or lectures hosted by health authorities on the proper disposal methods of expired medicines?	Yes	22	12.6	41	23.0	0.012
	No	153	87.4	137	77.0	
Attitude on which party should be responsible for the funding of the specialized centers	Health authorities	139	80.8	113	66.5	0.009
	Patients	2	1.20	5	2.90	
	Pharmaceutical companies	29	16.9	51	30.0	
	Community pharmacies	2	1.20	1	0.60	

A binary logistic regression analysis was conducted to identify factors associated with the outcome of the overall practice score (Table 8). The results showed that age and years of practice were the only significant predictors. Participants aged ≥30 years had significantly higher odds of the outcome compared to those aged <30 years (OR = 1.82, 95% CI: 1.05–3.14, p = 0.03). Similarly, participants with ≥5 years of practice were more likely to have the outcome than those with <5 years (OR = 1.84, 95% CI: 1.08–3.14, p = 0.03). In contrast, gender (OR = 1.03, p = 0.91), home residence (OR = 1.08, p = 0.80), and education level (OR = 0.74, p = 0.33) were not significantly associated with the outcome. Additionally, variables related to training and awareness—including attending courses or lectures (OR = 1.11, p = 0.15), perceived necessity of continuing education (OR = 0.79, p = 0.46), prior study of medication disposal during pharmacy education (OR = 1.17, p = 0.71), awareness of environmental risks (OR = 0.93, p = 0.76), and knowledge of disposal centers in Jordan (OR = 0.80, p = 0.40) did not show significant associations. Similarly, attitudes toward establishing specialized disposal centers (OR = 1.16, p = 0.66), willingness of community pharmacies to pay for disposal services (OR = 2.52, p = 0.12), and knowledge of how drug distributors dispose of expired medicines (OR = 1.20, p = 0.53) were not statistically significant predictors. Overall, the findings suggest that older age and longer professional experience are the main factors associated with the outcome, while demographic characteristics, educational background, and awareness-related variables do not appear to have a significant influence.

**Table 8:** Multiple predictor analysis of variables associated with good practice level.

Variable	Category Comparison	B	P-value	Odds Ratio (OR)	95% CI (Lower–Upper)	
Age	≥30 vs < 30 years	**0.60**	**0.03**	**1.82**	**1.05**	**3.14**
Gender	Female vs Male	0.03	0.91	1.03	0.66	1.61
Practice Years	≥5 vs < 5 years	**0.61**	**0.03**	**1.84**	**1.08**	**3.14**
Home Residence	Center vs Periphery (East/West)	0.07	0.80	1.08	0.65	1.78
Education Level	Bachelor’s vs Higher Education	−0.30	0.33	0.74	0.41	1.35
Have you ever attended courses or lectures	Yes vs No	0.11	0.15	1.11	0.96	1.29
Do you think there is an absolute necessity for community pharmacists to attend courses and lectures	Yes vs No	−0.23	0.46	0.79	0.43	1.48
Have you ever studied how to dispose of expired medicines during your years of studying pharmacy	Yes vs No	0.16	0.71	1.17	0.51	2.66
Are you aware of the dangers of disposing of medicines in improper methods to the environment	Yes vs No	−0.72	0.76	0.93	0.59	1.48
Did you know that there are any special centers for disposing of medicines in Jordan	Yes vs No	−0.22	0.40	0.80	0.48	1.34
Do you think Jordan needs a specialized center to dispose of medicines?	Yes vs No	0.15	0.66	1.16	0.59	2.28
Are community pharmacies willing to pay for the drug waste disposal service provided by this center?	Yes vs No	0.92	0.12	2.52	0.78	8.10
How do the drug distributors dispose of expired medicines after withdrawing them from pharmacies?	Do not know how vs other options	0.18	0.53	1.20	0.68	2.11

## Discussion

The handling of expired medicines by community pharmacists has gained interest in the literature [4,14,16,17,21,24,25]. Although few studies have evaluated community pharmacists’ knowledge, attitudes, and practices regarding the proper disposal of expired medicines in the Middle East, similar studies have been conducted in Kuwait, the UAE, Saudi Arabia, and Palestine [4,16,17,24].

The evaluation of community pharmacists’ disposal practices revealed that returning expired medicines to the pharmaceutical distributor is the most commonly adopted method, aligning with recommended guidelines. The practice score, calculated by summing adherence across five categories (solids, liquids, semisolids, Class B, and Class C), provided a comprehensive measure of overall compliance, with a maximum achievable score of 5.

The findings indicate that a slight majority of pharmacists (54.7%) demonstrated good practice, while 45.3% were classified as having poor practice. This suggests that although many pharmacists are aware of and implement proper disposal methods, a significant proportion still engage in suboptimal practices, particularly for liquids and semisolid formulations, where compliance rates were relatively lower. These dosage forms may present practical challenges in handling and storage prior to disposal, which could contribute to lower adherence.

In this study, the majority of community pharmacists (58.7–76.2%) reported returning expired medicines to pharmaceutical distributors, regardless of dosage form. Previous studies have reported various disposal methods, regardless of the dosage form of expired medicines [4,16,17,21,24,25]. In the UAE, most pharmacists returned expired medicines to contractors or distributors [17]. Additionally, 73.3% of community pharmacists in Palestine disposed of unwanted medicines by returning them to manufacturing companies and warehouses [4]. In Kuwait, the most prevalent disposal method was discarding expired medicines in trash cans, as reported by 73% of the pharmacists [16]. In Saudi Arabia, the most common disposal method was returning expired medicines to pharmaceutical distributors, as reported by >70% of pharmacists, regardless of dosage form [24]. In New Zealand, a related study found that the most common method of disposal for liquid dosage forms and class B controlled drugs was flushing them down the pharmacy sink [21]. Conversely, solid and semisolid forms were returned to the distributors [21]. Another similar study conducted in Anambra State, southeast Nigeria, showed that various disposal methods were used, such as rubbish bins, municipal waste, sink, toilet, National Agency for Food and Drug Administration and Control (NAFDAC) bins, pharmaceutical distributors, and burning, indicating no consistent disposal pattern among the respondents [25]. The similar disposal methods reported in Jordan, the UAE, Saudi Arabia, and Palestine, particularly the return of expired medicines to pharmaceutical distributors, highlight the vital role of distributors in managing the disposal of expired medicines from community pharmacies. Importantly, although some pharmacists reported that distributors dispose of expired medicines by flushing or garbage disposal prior to landfill, these methods are not recommended by the WHO and may contribute to environmental contamination. These findings reflect gaps in pharmacists’ knowledge regarding downstream disposal processes rather than confirmation of actual distributor practices. This highlights the need for improved transparency and regulatory oversight in pharmaceutical waste management.

Previous studies have shown that returning expired medicines to pharmaceutical distributors is the most common disposal method [4,16,17,21,24,25]. Therefore, this study assessed community pharmacists’ knowledge of how distributors handle and dispose of returned expired medicines. The results indicated that most community pharmacists in Jordan (75.4%) have limited knowledge of distributors’ strategies for handling and disposing of returned expired medicines. A study conducted in the UAE assessed pharmacists’ knowledge of how distributors handle returned expired medicines [17]. The results demonstrated that almost one-third of respondents did not know how distributors dispose of expired medicines. In contrast, the second third believed that expired medicines were incinerated, and the final third thought that expired medicines were discarded in the trash [17]. Additionally, pharmacists in New Zealand were uncertain about how contractors or distributors disposed of returned expired medicines, with some suggesting incineration [21]. Although most pharmacists in Nigeria chose the National Agency for Food and Drug Administration and Control (NAFDAC) as their primary disposal method, they were unaware of the actual disposal method NAFDAC used for returned expired medicines [25]. Nigerian pharmacists suggested that contractors dispose of expired medicines through incineration and other burning methods [25].

This study explored community pharmacists’ attitudes on the most effective strategies to reduce medicine expiration in their respective pharmacies. Participating pharmacists indicated that nearly expired medicines could be collected by medical representatives for return to distributors. Additionally, community pharmacists might limit stock quantities, collaborate with other pharmacies that need these medicines, or encourage physicians to prescribe generic names rather than brand names. Pharmacists in the UAE were also asked about the best strategies to minimize medicine expiration, with 51.7% reporting that stock limitation is the most effective strategy, and 48.3% indicating that collaboration with other pharmacies to manage nearly expired medicines is advisable [17]. The findings of our study and those of Kharaba et al. [17] suggested that pharmacists believe it is their responsibility to minimize the generation of expired medicines in their respective pharmacies.

The current study revealed that supplements, skincare, and hair products were the most frequently expired products in community pharmacies, according to 57.5% and 55.7% of community pharmacists, respectively. Similarly, Kharaba et al. [17] reported that the most expired products in community pharmacies in the UAE were skincare and hair care products, as well as supplements, including vitamins, minerals, and probiotics. These findings suggested that skincare and hair care products, which are also available in supermarkets and cosmetics stores at competitive prices, are particularly prone to expiration in community pharmacies.

Most community pharmacists in Amman (70.3%) were aware of the harmful effects of improper disposal of expired medicines on the environment. This aligns with Alghadeer and Al-Arifi [24] and Abahussain et al. [16], who reported that 80% and 82% of participating pharmacists in Saudi Arabia and Kuwait, respectively, recognized the potential environmental hazards associated with the improper disposal of expired medicines. Additionally, Abahussain et al. [16] reported that 97% of pharmacists agreed that it is their responsibility to protect the environment from expired medicine hazards by collecting expired medicines. Moreover, Nairat et al. [4] showed that 61.3% of pharmacists agreed and 26% strongly agreed that the improper disposal of unwanted medicines negatively impacts the environment.

To raise awareness of the proper disposal of expired medicines, this study evaluated whether pharmacists had studied proper disposal methods during their pharmacy education. In their responses, most community pharmacists reported not having received any courses on the proper disposal of expired medicines in their pharmacy school curricula, nor having attended lectures or workshops organized by health authorities. Additionally, they acknowledged the need to participate in future lectures and workshops focused on the proper disposal of expired medicines. Therefore, it has been shown that taking courses, attending workshops or seminars, or undergoing undergraduate training that covers the safe disposal of expired medicines will raise community pharmacists’ awareness of proper disposal methods of expired medicines [4,17,24,25].

In this study, community pharmacists showed a strong demand for a specialized disposal center for expired medicines. Our findings aligned with those reported by Kharaba et al. [17], who found that 68.4% of participating pharmacists in the UAE expressed the need for such a center, which the Ministry of Health should primarily fund. Additionally, 97.3% of community pharmacists supported establishing a drug-disposal system in Palestine. Furthermore, 90% of respondents in New Zealand agreed that health authorities should establish a system for the disposal of unwanted medicines [21].

Finally, this study demonstrated significant correlations between community pharmacists’ knowledge, attitudes, and practices regarding the disposal of expired medicines in Jordan and their responses to the questionnaire. For instance, the age and years of experience of community pharmacists influenced their responses toward the disposal of expired medicines (p < 0.05), the reduction of expired medicines in community pharmacies (p < 0.05), and attendance of workshops and lectures on the proper disposal of expired medicines organized by health authorities (p < 0.05). Additionally, the gender of community pharmacists influenced their perceptions of the need for a specialized center in Jordan for the disposal of expired medicines and of the necessity to establish one (p < 0.05). The correlations between the study variables and demographic data, as well as the pharmacists’ knowledge, attitudes, and practices in the UAE and Palestine, were also examined, yielding similar results [4,17].

### Research limitations

This study has several limitations that should be considered when interpreting the findings. First, the use of a snowball sampling technique may have introduced selection bias, as participation relied on professional networks and voluntary referral. Consequently, pharmacists who were more professionally engaged or connected may have been more likely to participate. This sampling approach limits the generalizability of the findings to all community pharmacists in Jordan. Second, data were collected via self-reported questionnaires, which are susceptible to social desirability bias and recall bias. Participants may have over-reported environmentally responsible practices or under-reported improper disposal behaviors. Third, the use of mixed data-collection modes (paper-based and online questionnaires) may have introduced potential mode effects. Differences in the survey environment or perceived anonymity between paper and online responses could have influenced participants’ answers. Fourth, the cross-sectional study design precludes causal inference. Although associations between demographic variables and knowledge, attitudes, and practices were identified, no conclusions can be drawn regarding causality. Finally, multiple Chi-square tests were conducted to examine associations between demographic variables and questionnaire responses. Conducting multiple statistical tests increases the risk of Type I error. To mitigate this risk, analyses were limited to predefined key hypotheses, and statistical significance was interpreted cautiously.

## Conclusion

Overall, the findings showed that most participating community pharmacists were aware of the environmental risks associated with the improper disposal of expired medicines. However, this awareness was not consistently translated into optimal practice. Although more than half of pharmacists (54.7%) demonstrated good disposal practices based on the composite practice score, a considerable proportion (45.3%) still engaged in poor practices. Notably, gaps were particularly evident in the disposal of liquid and semisolid dosage forms, highlighting specific areas that require targeted improvement. The study also revealed that age and professional experience were the only significant predictors of good practice. Pharmacists aged 30 years or older and those with at least five years of experience were more likely to demonstrate proper disposal practices. In contrast, gender, education level, training, and awareness-related factors did not significantly influence practice, suggesting that experience-based learning may play a more critical role than formal education or perceived knowledge alone. These findings underscore the need for more effective strategies to bridge the gap between awareness and practice. Enhancing the role of community pharmacists in the proper disposal of expired medicines requires structured interventions, such as organizing targeted workshops and continuous professional development programs led by health authorities, and strengthening pharmacy curricula to include practical training on medication disposal. Furthermore, establishing specialized centers for the safe disposal of expired medicines in Jordan remains an urgent priority. Regulatory authorities should also implement and enforce stricter policies to ensure compliance with proper disposal practices in community pharmacies. Additionally, improving stock management practices within pharmacies could help reduce the accumulation of expired medicines, thereby decreasing the burden of improper disposal. In conclusion, while community pharmacists show a reasonable level of awareness and moderate adherence to proper disposal methods, significant gaps persist. Addressing these gaps through policy enforcement, education, and infrastructure development is essential to promote environmentally safe disposal methods and protect public health.

## Supporting information

S1 Table Disposal methods of dosage forms as reported by the World Health Organization [7]. (DOCX)

S2 Table Questionnaire questions. (DOCX)
