# Supplementary material for: Disposal of expired medicines in Jordan: Practices of community pharmacists
Source: PLoS One. 2026 May 15;21(5):e0348951. doi: 10.1371/journal.pone.0348951 (PMC13178985; doi:10.1371/journal.pone.0348951)
Supplement: S1 Table — (DOCX) [file pone.0348951.s001.docx]

**S1 Table: Disposal methods of dosage forms as reported by the World Health Organization [1]**.

| **Category** | **Disposal methods** | **Comments** |
| --- | --- | --- |
| **Solids** | - Landfill | - No more than 1% of the daily municipal waste should be disposed of in a landfill in an untreated (non-immobilized) form. |
| **Semi-solids** | - Waste encapsulation |  |
| **Powders** | - Waste inertization - Medium- and high-temperature incineration (cement kiln incinerator) |  |
| **Liquids** | - Sewer - High-temperature incineration (cement kiln incinerator) | - Antineoplastics are not disposed of in the sewer. |
| **Controlled drugs** | - Waste encapsulation - Waste inertization - Medium- and high-temperature incineration (cement kiln incinerator) | - Not to landfill unless encapsulated. |

1. WHO. Guidelines for the safe disposal of expired drugs: World Health Organization; 2006 [Available from: <https://www.emro.who.int/images/stories/pakistan/documents/pak_documents/Guidelines_for_Expired_Medicines.pdf>.
