# Supplementary material for: Disposal of expired medicines in Jordan: Practices of community pharmacists
Source: PLoS One. 2026 May 15;21(5):e0348951. doi: 10.1371/journal.pone.0348951 (PMC13178985; doi:10.1371/journal.pone.0348951)
Supplement: S2 Table — (DOCX) [file pone.0348951.s002.docx]

**S2 Table**: **Questionnaire questions with items labeled to correspond to knowledge, attitude, and practice.**

| **Questions** | **Answers** | | | | **Domain** | | |  |
| --- | --- | --- | --- | --- | --- | --- | --- | --- |
| **Section 1: Demographic Data of Participants** | | | | | Not applicable | | |  |
| 1-To which age group do you belong? | <30 | | | |  | | |  |
|  | 30-39 | | | | | |  |  |
|  | 40-49 | | | | | |  |  |
|  | 50-59 | | | | | |  |  |
|  | ≥60 | | | | | |  |  |
| 2- Gender | Male | Not applicable | | | | | |  |
|  | Female |  | | | | | |  |
| 3- How long have you been practicing Pharmacy as a pharmacist in a community pharmacy? | <1 year | Not applicable | | | | | |  |
|  | 1-4 years | | | | | |  |  |
|  | 5-9 years | | | | | |  |  |
|  | ≥10 years | | | | | |  |  |
| 4- In which part of the capital, Amman, is your pharmacy located? | Center | | Not applicable | | | | |  |
|  | East | | | | | |  |  |
|  | West | | | | | |  |  |
| 5-Educational Degree | Bachelor's Degree in Pharmacy | | | Not applicable | | | | |
|  | Doctor of Pharmacy (Pharm D) | | | | |  | | |
|  | Master’s degree | | | | | |  |  |
|  | Doctor of Philosophy (PhD) | | | | | |  |  |
| **Section 2: Disposal Methods of Expired Medicines** | | | | | | |  |  |
| 6- In your pharmacy, most chronic medications are purchased from | Agent | | | | | | Knowledge |  |
|  | Subagent | | | | | |  |  |
| 7- In your pharmacy, over-the-counter medications (such as mild analgesics, such as paracetamol) are purchased from | Agent | | | | | | Knowledge |  |
|  | Subagent | | | | | |  |  |
| 8- In your pharmacy, controlled drugs of categories B (such as codeine) and C (such as diazepam) are purchased from | Agent | | | | | | Knowledge |  |
|  | Subagent | | | | | |  |  |
| 9- How to dispose of expired solid medicines (pills, capsules, suppositories, etc.) in your pharmacy | In the rubbish bins | | | | | | Practice |  |
|  | Throwing them down the sink | | | | | |  |  |
|  | Flushing them down the toilet | | | | | |  |  |
|  | Return to the pharmaceutical distributor. | | | | | |  |  |
|  | other | | | | | |  |  |
| 10- How to dispose of expired liquid medicines (suspension solutions, injection solutions, etc.) in your pharmacy | In the rubbish bins | | | | | | Practice |  |
|  | Throwing them down the sink | | | | | |  |  |
|  | Flushing them down the toilet | | | | | | Practice |  |
|  | Return to the pharmaceutical distributor. | | | | | |  |  |
|  | Don’t know | | | | | |  |  |
| 11- How to dispose of expired semi-solid medicines (creams, ointments, etc.) in your pharmacy | In the rubbish bins | | | | | | Practice |  |
|  | Throwing them down the sink | | | | | |  |  |
|  | Flushing them down the toilet | | | | | |  |  |
|  | Return to the pharmaceutical distributor. | | | | | |  |  |
|  | Don’t know | | | | | |  |  |
| 12- How to dispose of expired Class B controlled drugs (such as codeine) in your pharmacy | In the rubbish bins | | | | | | Practice |  |
|  | Throwing them down the sink | | | | | |  |  |
|  | Flushing them down the toilet | | | | | |  |  |
|  | Return to the pharmaceutical distributor. | | | | | |  |  |
|  | Don’t know | | | | | |  |  |
| 13- How to dispose of expired Class C controlled drugs (such as diazepam) in your pharmacy | In the rubbish bins | | | | | | Practice |  |
|  | Throwing them down the sink | | | | | |  |  |
|  | Flushing them down the toilet | | | | | |  |  |
|  | Return to the pharmaceutical distributor. | | | | | |  |  |
|  | Don’t know | | | | | |  |  |
| 14- How do the drug distributors dispose of expired medicines after withdrawing them from pharmacies | Placing medicines in the garbage before disposing of them in a landfill | | | | | | Knowledge |  |
|  | Burning medicines in designated places | | | | | |  |  |
|  | By throwing it in the toilet | | | | | |  |  |
|  | By throwing them in the sink | | | | | |  |  |
|  | I don’t know. I’ve never been told how | | | | | |  |  |
|  | Other | | | | | |  |  |
| 15- Have you ever attended courses or lectures held by health authorities on the proper disposal methods of expired medicines? | Yes | | | | | | Attitude |  |
|  | No | | | | | |  |  |
| 16- Do you think there is an absolute necessity for community pharmacists to attend courses and lectures that the competent health authorities hold on the proper disposal methods of expired medicines? | Yes | | | | | | Attitude |  |
|  | No | | | | | |  |  |
| 17- Have you ever studied how to dispose of expired medicines during your years of studying pharmacy? | Yes | | | | | | Attitude |  |
|  | No | | | | | |  |  |
| 18- Are you aware of the dangers of improper disposal of expired medicines to the environment? | Yes | | | | | | Knowledge |  |
|  | No | | | | | |  |  |
| **Section 3: Future handling of expired medicines** | | | | | | |  |  |
| 19-Did you know there are any special centers for disposing of medicines in Jordan? | Yes | | | | | | Knowledge |  |
|  | No | | | | | |  |  |
| 20- Do you think Jordan needs a specialized center to dispose of expired medicines? | Yes | | | | | | Attitude |  |
|  | No | | | | | |  |  |
| 21- The funding of this center must be through: | Health authorities | | | | | | Attitude |  |
|  | Patients | | | | | |  |  |
|  | Pharmaceutical companies | | | | | |  |  |
|  | Community pharmacies | | | | | |  |  |
| 22—Are community pharmacies willing to pay for the drug waste disposal service provided by this center? | Yes | | | | | | Attitude |  |
|  | No | | | | | |  |  |
| **Section 4: How to reduce the number of EMs in community pharmacies** | | | | | | |  |  |
| 23- Actions for managing (reducing) quantities of expired medicines: | Stock limitations | | | | | | Practice |  |
|  | Cooperation with other pharmacies to exchange almost expired medicines | | | | | |  |  |
|  | Recall of medicines by distributors and resupply of pharmacies in dire need of almost-expired medicines | | | | | |  |  |
|  | Other | | | | | |  |  |
| 24- Please tick the most common EMs in your community pharmacy in the last 24 months | Skincare and hair products | | | | | |  |  |
|  | Supplements: vitamins, minerals, and probiotics | | | | | |  |  |
|  | Analgesics and antipyretic | | | | | |  |  |
|  | Antibiotics | | | | | |  |  |
|  | Oral care products | | | | | |  |  |
|  | Antidiabetic drugs | | | | | |  |  |
|  | Asthma medications | | | | | |  |  |
|  | Fertility medications (contraceptives and hormones) | | | | | |  |  |
|  | Nasal decongestants | | | | | |  |  |
|  | Antihypertensive agents | | | | | |  |  |
|  | Antacids | | | | | |  |  |
|  | Baby care products | | | | | |  |  |
|  | Antihistamine drugs | | | | | |  |  |
|  | Antihyperlipidemic agents | | | | | |  |  |
|  | Eye drops | | | | | |  |  |
|  | Antispasmodics | | | | | |  |  |
|  | Antiepileptics | | | | | |  |  |
|  | Thyroid disorder medications | | | | | |  |  |
|  | Cardiology medications | | | | | |  |  |
|  | Wound healing products | | | | | |  |  |
|  | Weight loss medications | | | | | |  |  |
|  | Motion sickness medications | | | | | |  |  |
|  | Laxatives | | | | | |  |  |
|  | Nicotine replacement products | | | | | |  |  |
|  | Anticoagulants | | | | | |  |  |
